# Presidential address: reflection on work from April 2019 to 2022 and appreciation to the staff and volunteers

**DOI:** 10.3352/jeehp.2022.19.39

**Published:** 2022-12-30

**Authors:** Yoon-Seong Lee

**Affiliations:** President, Korea Health Personnel Licensing Examination Institute, Seoul, Korea; Hallym University, Korea

On December 21, 2022, the new 9th president of the Korea Health Personnel Licensing Examination Institute (KHPLEI) was elected at the executive board meeting of KHPLEI in Seoul. I was delighted by the election results since a new and younger president will lead the KHPLEI starting in February 2023, and I hope that the KHPLEI will be more dynamic and forward-looking. Before ending my devoted service as the president of the KHPLEI, which has lasted since April 22, 2019, I would like to reflect on the work we at the KHPLEI have accomplished during my term.

## Accomplishment of tasks announced at the inaugural speech in June 2019

When I began the president’s job, I announced the following plans [1]: first, training experts in making high-quality test items; second, strengthening work at the research and development headquarters; third, providing more convenient services for examinees, including a computer-based testing (CBT); fourth, aiding foreign countries’ institutes in their management of licensure; fifth, adopting a clinical skills examination as part of the Korean Dental Licensing Examination, which will take effect in the second half of 2021; and sixth, widely including ethics items in various licensing examinations.

Most of these plans were successfully carried out. Many item-making experts were trained at the test construction center. As for research activities, 2 papers on cut scores were published by the research and development headquarters staff in the KHPLEI’s official journal. The cut score does not change according to the number of items using different standard-setting methods [2]. This result provides evidence to use a portion of items for standard-setting. If all the items would be used, too much time or workforce would be required. Another study related to the possible adoption of the Angoff method for the Korean Nursing Licensing Examination [3]. The procedural validity of panelists was acceptable, as shown by a high confidence level. These results show that determining cut scores by an expert panel’s determination is practical.

CBT was successfully executed for the Korean Medical Licensing Examination in January 2022 [4]. It will be a priming event for other professionals to adopt CBT. As for aiding foreign institutes with the same aims and scope, cooperation with the corresponding institute in Vietnam was not as rapid as could have been hoped due to the coronavirus disease 2019 (COVID-19) lockdown, and the exchange of human resources became difficult starting in early 2020. More proactive collaboration is expected as the COVID-19 pandemic situation stabilizes. As announced, a clinical skills examination was introduced in the Korean Dental Licensing Examination in 2021. The number of ethics items increased from 26 in 6 licensing examinations in 2018 to 37 in 12 licensing examinations in 2020 (KHPLEI data not published).

## Adoption of computer-based testing in all professionals’ licensing examination

Starting in 2023, CBT will be implemented in the Korean Dental Licensing Examination, Korean Oriental Doctor Licensing Examination, and Korean Care Worker Licensing Examination.

## High level of evaluation record of management performance

The Korean government’s Ministry of Health and Social Welfare has regularly evaluated the performance of public institutes, including KHPLEI, under the supervision of the Ministry. The measurement items in 2020 included the following: management strategy and leadership, social value implementation, budget management, welfare management, innovation and communication, major implementation projects, and quarantine actions against the COVID-19 pandemic. The KHPLEI received the best record in this evaluation system in 2020. This was possible owing to all staff members’ hard work. In particular, the quarantine guidelines to protect examinees from COVID-19 were well kept at the examination sites.

## Appreciation to staff and volunteers for their devotion

The KHPLEI’s performance during the term starting in April 2019 would have been impossible without the staff and volunteers’ devotion. Many professors and experts in 24 health professions worked to produce high-quality licensing examination items and user-friendly implementations of the examinations. Furthermore, new technologies have been introduced without difficulties with their help. The KHPLEI is not merely operated by staff, but a place where experts in all health fields reach consensus. I believe that new and innovative activities will continue to take place under the leadership of the new president. I am happy with and proud of the staff and volunteers. Additionally, the promotion of the official journal, *Journal of Educational Evaluation for Health Professions*, as an invaluable information delivery vehicle for licensing examinations is a great pleasure. I appreciate the staff and volunteers again and end with a new year’s greeting to readers, reviewers, and authors: Happy New Year, and I hope that we will all be free from the COVID-19 pandemic.
